# Surgical Management of Lumbar Hardware Failure Due to Recurrent Postoperative Spondylodiscitis: Case Report

**DOI:** 10.7759/cureus.27457

**Published:** 2022-07-29

**Authors:** Edwin Bernard, Brenda Enelis, Renat Nurmukhametov, Manuel de Jesus Encarnacion Ramirez, Medet Dosanov, Ilya Shirshov, Ibrahim E Efe, Issael Jesus Ramirez Pena, Rossi E Barrientos Castillo

**Affiliations:** 1 Division of Spine Surgery, Central Clinical Hospital of the Russian Academy of Sciences, Moscow, RUS; 2 Neurological Surgery, Peoples' Friendship University of Russia, Moscow, RUS; 3 Neurosurgery, Medical System (MEDSI) Clinical Hospital, Moscow, RUS; 4 Neurological Surgery, Charité - Universitätsmedizin, Berlin, DEU; 5 Neurosurgery Oncology, Fellow Royal Melbourne Hospital, Melbourne, AUS; 6 Neurological Surgery, Peoples Friendship University of Russia, Moscow, RUS

**Keywords:** 360o spine surgery, incidence, debridement, alif, tlif, recurrent, infection, s: spondylodiscitis

## Abstract

Spondylodiscitis is a rare bacterial infection of the vertebrae and intervertebral discs that causes inflammation and follows a destructive course. When conservative management fails, surgical management requires immediate debridement of the infective focus, with decompression and stabilization through a ventral approach. The most frequently involved locations are the lumbar spine (58%), thoracic (30%), and cervical (11%) regions. Gram-positive organisms such as *Staphylococcus aureus* and Streptococcus species are the most commonly isolated organisms (67% and 24%, respectively). Pathophysiologically, infectious spondylodiscitis begins in the anterior portion of the vertebral body, due to its rich vascular supply, and then spreads to the rest of the vertebral body and along the medullary spaces. In this study, we report the management of recurrent lumbar postoperative spondylodiscitis with transforaminal lumbar interbody fusion (TLIF) hardware failure in a 62-year-old female.

## Introduction

Spondylodiscitis is a rare bacterial infection of the vertebra and intervertebral discs that follows an inflammatory and destructive course. Postoperative spondylodiscitis is a rare but serious complication after lumbar disc surgery [1]. There are various predisposing factors that may increase the risk of discitis, such as the spinal vascular system, degenerative osteoarthritis disease, spinal trauma, and genital or urinary infection. Also, it can be secondary to direct inoculation by spinal surgery or penetrating trauma. When conservative management fails, spondylodiscitis requires immediate debridement of the focus with decompression and stabilization through a ventral approach [2]. Otherwise, severe complications may occur, such as sepsis, destruction of the vertebral body, abscess, or neurological deficits [3]. Discitis typically arises as a result of hematogenous spread. Pyogenic infections most frequently involve the lumbar spine (58%), the thoracic (30%), and cervical (11%) regions. Gram-positive organisms such as *Staphylococcus aureus* and Streptococcus species are the most commonly isolated organisms (67% and 24% of cases, respectively) [4,5]. The incidence of spondylodiscitis after conventional or microscopic surgery varies between 0.1% and 3% [6]. Prophylactic antibiotics may be considered to decrease the rate of infections in patients with instrumented spine fusion [7]. The use of antibiotics has proven to reduce the incidence of postoperative discitis. Vertebral infection can be acquired in many ways, including hematogenous or infected surgical wounds in micro and conventional open procedures [8,9]. Pathophysiologically, infectious spondylodiscitis begins in the anterior portion of the vertebral body, due to its rich vascular supply, and then spreads to the rest of the vertebral body along the medullary spaces [10].

## Case presentation

Materials and methods

**Figure 1:**
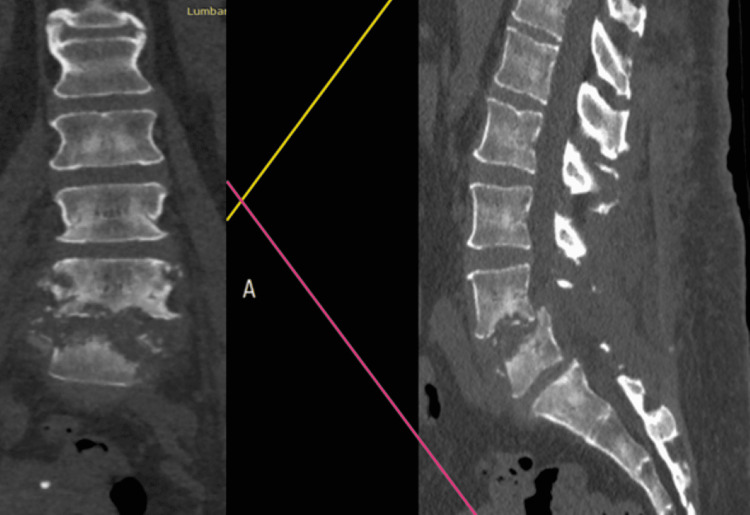
Preoperative of the first surgical stage, from left to right: coronal and sagittal CT slides. T1 showing a destructive process both (L4-L5) endplates and vertebral bodies destruction, starting from the ventral portions of both structures, anterolisthesis of L4 is also observed with stenosis L4-L5.

**Figure 2:**
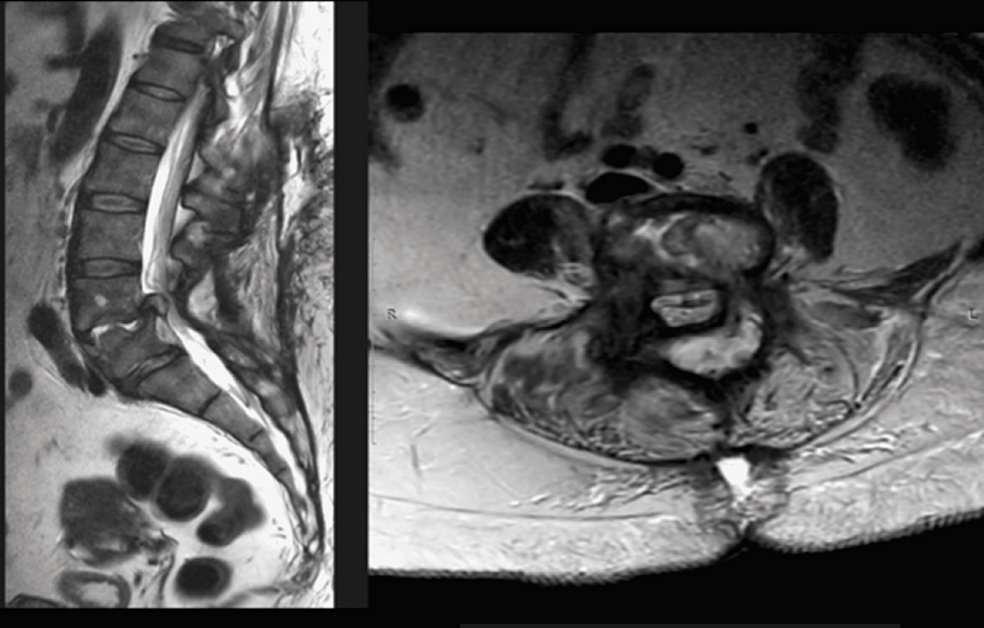
Preoperative of the first surgical stage, from left to right: sagital and axial MRI slides on T2. It is observed a remarkable stenosis narrowing and compressing the spinal canal, and stenosis and the infectious process extending from the ventral part of the vertebral body at the level L4-L5.

The patient’s record and radiological images (Figures 1-2) were carefully analyzed before booking the procedure. Our female patient underwent an anterior lumbar interbody fusion (ALIF; surgical approach consisted of an incision on the left anterior-lateral surface of the abdominal skin, at the height of the L4-L5 vertebrae previously determined by fluoroscopy; a retroperitoneal approach was performed to access the level). Two hours after the first surgical time, a second surgical time was performed as a transforaminal lumbar interbody fusion (TLIF) with 5-inch paraspinal incisions via a standard open approach. Surgical indications included spondylolisthesis, hardware failure with vertebral bone destruction and anterolisthesis of L4 vertebrae, recurrent herniated intersomatic cage, and adjacent level disease. The procedure was performed by the chairman of the department. The patient showed radiological signs of vertebral osteolysis in the anterior and middle third of L4 and L5.

Instrumentation

The patient had bilateral posterior pedicle screw-rod Pathfinder titanium implants (Abbott Spine, Austin, TX). They were replaced by the hardware-tipped Stryker XIA 3 pedicle system: pedicle screw with nut 6.0 mm × 40 mm 2 pcs, 7.0 mm × 45 mm 2 pcs, 6.0 mm × 45 mm 2 pcs rod - 1 pc. cylindrical titanium MESH cage, interbody cage mmgr - 1 pc graft.

Surgical techniques description

First Surgical (ALIF)

The patient was positioned in a prone position. General anesthesia with tracheal intubation and mechanical ventilation. A skin incision was made along the anterior-lateral surface of the abdomen on the left, up to 15 cm long in the projection of the L4-L5 vertebrae. Then, in the retroperitoneal part of the approach on the left, we found some technical difficulties (the exfoliated peritoneum was injured in several places because it was fibrosed as a result of the previous surgery, then was sutured, and the vertebral bodies were injured and retracted with meter-long napkins to the right), the L4-L5 intervertebral disc was reached (the main vessels were retracted to the right, the segmental vessels were ligated). There was a fibrosis process in the retroperitoneal space and a pronounced scarring process in the region of the vertebral bodies, with adhesions to the parietal peritoneum. The culture sample was taken from the intervertebral disc L4-L5. Under a magnifying technique of 2.5×, anterior decompression of the dural sac and roots was formed using microsurgical instruments. During the revision, the spinal canal was free; there was no pressure on the dural sac, passing and exiting roots. With the help of interbody distractors, the titanium MESH cage was selected according to its size. The autologous bone of the iliac crest was taken from a separate access in the left iliac region. The autologous bone from the iliac crest is placed into the titanium MESH cage, which is installed in the interbody space L4-L5. X-ray control shows the implant is stable, installed with active drainage, layered wound closure, and aseptic bandage.

Second Surgical Time (TLIF)

The surgery was performed with the patient in a prone position. A posterior median incision of 12 cm was made in the projection of the spinous processes L3-L4-S1 along the postoperative scar. The spinous processes, arches, and articular processes are skeletonized. Hemostasis was performed.

**Figure 3:**
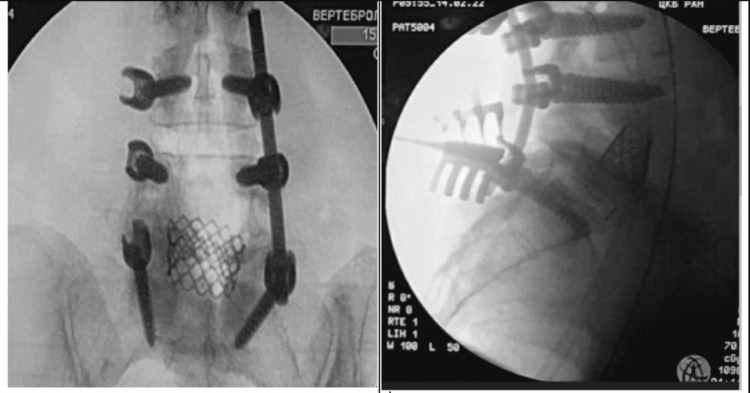
From left to right: intraoperative AP fluoroscopy control and intraoperatory lateral control. AP view showing the MESH implant (previously placed during the ALIF) at level L4-L5 and, the transpedicular screws L3-S1 (at level L5 no screw was placed). Intraoperatory lateral control, it is observed that the interbody cage (L5-S1) is in right position and stable, also the MESH implant (L4-L5) and the screws are displayed to be in the correct position.

Under X-ray control, pedicle screws were introduced bilaterally into the vertebrae L3-L4-S1 (6.0 mm × 40 mm 2 pcs, 7.0 mm × 45 mm 2 pcs, 6.0 mm × 45 mm 2 pcs). Under a magnifying technique of 2.5x using microsurgical instruments, medial facetectomy, interlaminectomy, foraminal decompression of L5-S1 on the right, and decompression on the contralateral side were performed. The intervertebral space was treated to place the intersomatic cage 10 mm, 4 gr (1 pc), installed with autobone, and verified that the cage is stable (Figure 3).

During the revision, the canal was free and there was no sign of pressure on the dural sac. Hemostasis was performed. The rods (5.5 mm × 50 mm) were contoured for physiological curves, the screws were fixed, and the revision drain was placed according to Ridon. The wound was sutured in layers. An aseptic bandage was placed on the skin, previously sutured with nylon monofilament 3.0. Hardware used: Stryker XIA 3 pedicle system; pedicle screw with nut 6.0 mm × 40 mm 2 pcs, 7.0 mm × 45 mm 2 pcs, 6.0 mm × 45 mm 2 pcs rod - 1 pc cage, cylindrical titanium MESH cage, interbody cage Stryker OIC 10 mm 4 gr - 1 pc.

Case presentation

A 62-year-old female presented with a rich past medical history of hypertension grade 3 (AG-1, risk factor-3), heart failure, atherosclerosis of the aorta, diffuse small focal cardiosclerosis, and nodular goiter. The patient also had medically compensated hypothyroidism and chronic gastritis. Past surgeries: operation in 1992, type cholecystectomy; removal of a hernia of the anterior abdominal wall in 2006; iron deficiency anemia of moderate severity. The patient came to our department after two recent spinal interventions: L4-L5 TLIF surgery that was performed three months before, on day 27 after the first surgery, during the control appointment, was found to have an implant infection with a subsequent hardware failure and instability showing canal stenosis due to cage migration at the level of the intervention (L4-L5); a planned reintervention surgery was performed with the removal of metal fixators, revision, and with due asepsis and antisepsis of the intervention area. The patient was discharged home two weeks with remarkable recovery after revision surgery; however, it was 10 days before our follow-up consultation she began complaining of mechanical lumbar pain and radiculopathy with progressive deterioration of her motor strength, referring to her inability to stand on her own. The neurological exam showed weakness bilateral in lower limbs (3/5 left side and 4/5 right side of the Daniels scale), decreased tendon reflexes, more markedly on the left side, Lassegue 35 degrees left side and 50 degrees right side, and lumbar muscular spasm and tenderness at the levels L3-S1. The surgical wound was clean, dry, and cicatrized. Initial MRI imaging confirmed sagittal vertebral instability with bone destruction in both L4 and L5 vertebral bodies and anterolisthesis of L4. After a multidisciplinary discussion, it was decided to perform a 360-degree intervention divided into two surgical times.

Postoperative care

Mobilization began 24 hours after the procedure when the patient showed very good improvement. On postoperative day 15, the patient was discharged to her home. Once the causal infectious agent is identified in the histopathological test results, Staphylococcus aureus, our approach is directed to pharmacological therapy for three months. Our recommendations included: walking to tolerance periodically, swimming, good eating habits, and weight loss to reduce mechanical stress. Heavy lifting and other strenuous activities are discouraged for at least six months. Six weeks postoperatively, the patient was otherwise permitted to resume her normal life as soon as she could tolerate it, following all the recommendations given before discharge.

The postoperative diagnosis of spondylodiscitis was confirmed thanks to bacteriologic and hematologic laboratory results. Blood cultures revealed Streptococcus aureus as the responsible pathogenic microorganism. The patient was able to completely go back to normal life after three months.

## Discussion

It is well known that spinal inflammatory processes can generally affect the dorsal and lumbar regions; symptoms are usually progressive and can often be characterized by fever, chills, night sweats, and weight loss also can be present with neurological compromise [1,11,12]. Laboratory results are usually abnormal but can also be normal.

Usually, the starting point is the anterior third of the vertebral body, where the infection has a high-rate capacity of spreading due to its great vascularity. It is clear that the definition of "hardware failure" in clinical situations such as the one we are presenting does not refer to a malfunction due to a hardware defect. Rather, it is a failure induced by a cascade of pathophysiological mechanisms that have a direct influence on the mechanics of movement and the integrity of the affected segment, which leads to the destruction of bone tissue and, consequently, to the failure of the fixation material in the absence of an appropriate biomechanical support point. Alloy corrosion and wear are a significant concern in orthopedic applications. Mechanical forces applied to spinal constructs enhance these processes. Hence, their degradation takes numerous forms, with the most commonly accepted modes being fretting wear, fatigue corrosion, as well as protein and cellular degradation.

## Conclusions

Hardware failure is a rare complication. More studies would be necessary in order to demonstrate and control the factors that lead to hardware failure. Since we do not have a large number of studies on hardware failure, however, it is considered to lead to a number of complications that can compromise the patient's life. Spondylodiscitis involves the vertebral bodies and the intervertebral disc and may involve the paravertebral structures and the spinal canal. MRI continues to be the method of choice for the diagnosis of spondylodiscitis, given its specificity for soft tissue.

Early diagnosis and treatment are of vital importance. Untreated spondylodiscitis can take an aggressive course that puts the patient's life at risk. It is always necessary to see the patient from the clinical point of view before the surgical one to avoid making hasty surgical decisions that, more than helping, could complicate the patient's clinical status. The identification of the pathogenic agent is one of the most important points to ensure effective results. The sooner we start intravenous therapy directed at the specific agent, the better results we will obtain in the short term and the better the prognosis of the patient.
